# Monitoring of heat-induced carcinogenic compounds (3-monochloropropane-1,2-diol esters and glycidyl esters) in fries

**DOI:** 10.1038/s41598-020-72118-z

**Published:** 2020-09-15

**Authors:** Yu Hua Wong, Kok Ming Goh, Kar Lin Nyam, Ling Zhi Cheong, Yong Wang, Imededdine Arbi Nehdi, Lamjed Mansour, Chin Ping Tan

**Affiliations:** 1 Department of Food Technology, Faculty of Food Science and Technology, Universiti Putra Malaysia, 43300 Seri Kembangan, Selangor Malaysia; 2 Department of Food Science and Nutrition, Faculty of Applied Sciences, UCSI University, 56000 Cheras, Kuala Lumpur Malaysia; 3 Department of Food Safety and Quality, School of Marine Science, Ningbo University, Ningbo, 315211 China; 4 JNU-UPM International Joint Laboratory on Plant Oil Processing and Safety (POPS), Department of Food Science and Engineering, Jinan University, Guangzhou, 510632 China; 5 Chemistry Department, College of Science, King Saud University, P.O. BOX 2455, Riyadh, 11451 Saudi Arabia; 6 Chemistry Department, El Manar Preparatory Institute for Engineering Studies, Tunis El Manar University, P.O. Box 244, 2092 Tunis, Tunisia; 7 Zoology Department, College of Science, King Saud University, Saudi Arabia, P.O. Box 2455, Riyadh, 11451 Saudi Arabia

**Keywords:** Chemistry, Materials science

## Abstract

3-Monochloropropane-1,2-diol (3-MCPD) esters and glycidyl esters (GE) are heat-induced contaminants which form during oil refining process, particularly at the high temperature deodorization stage. It is worth to investigate the content of 3-MCPD and GE in fries which also involved high temperature. The content of 3-MCPD esters and GE were monitored in fries. The factors that been chosen were temperature and duration of frying, and different concentration of salt (NaCl). The results in our study showed that the effect was in the order of concentration of sodium chloride < frying duration < frying temperature. The content of 3-MCPD esters was significantly increased whereas GE was significantly decreased, when prolong the frying duration. A high temperature results in a high 3-MCPD ester level but a low GE level in fries. The present of salt had contributed significant influence to the generation of 3-MCPD. The soaking of potato chips in salt showed no significant effect on the level of GE during the frying. The oil oxidation tests showed that all the fries were below the safety limit. Hence, the frying cycle, temperature and the added salt to carbohydrate-based food during frying should be monitored.

## Introduction

Deep-fat frying is commonly being used to process food. During the process, heat transfer between the fried food and oil is occurs. The frying oil replaced the moisture in food during the dehydration process. Thus, the frying oil quality is highly influencing the fried food^1^. During deep-fat frying, free fatty acids, aldehydes, ketones, hydrocarbons, diacylglycerol and monoacylglycerol are formed from numerous complex interrelation reactions induced by hydrolysis, oxidation, isomerization and polymerization^2^. Palm olein is widely being used for deep-fat frying because of the stability of the oil and high smoke point. This is due to the unique properties of palm olein, which contain the relative balance of unsaturated and saturated fatty acids^3^. It is suitable for repeated frying within a certain number of frying cycles.

3-Monochloropropane-1,2-diol (3-MCPD) esters and glycidyl esters (GE) are trace contaminants formed during the high-temperature refining of oils, especially during the deodorization process^4,5^. The temperature for deep-fat frying usually is within 150–190 °C^6^, which fall within the temperature range of refining process. Researchers believe that the 3-MCPD esters are from the conversation of triacylglycerol to cyclic acyloxonium, together with chloride ions^7^. 3-Monochloropropane-1,2-diol is in vivo carcinogenic and in vitro genotoxic^8^. Glycidyl esters are suggested to be formed through an intermediate stage during 3-MCPD ester formation via monoacylglycerol-derived acyloxonium ion pathway^9^. The free form of glycidyl is a carcinogenic directly acting multisite mutagen in animal studies that reacts readily with cellular nucleophiles.

Frying oils that are transferred into the fried potatoes can be the main sources of 3-MCPD esters in fried potatoes^10^. A potato, which contains carbohydrates, protein, fat, vitamins and other minor constituents, can undergo a very complex chemical reaction with salt under prolonged high-temperature deep frying^11^. Therefore, the influence of the frying temperature, frying time and salt was monitored. The concentration of salt used for soaking were from 1 to 5%. The chosen temperature were 160 °C and 180 °C, and continue the frying for 5 days. The parameters chosen were to simulate the most common frying condition.

## Results

### Influence of the temperature during frying

**Figure 1:**
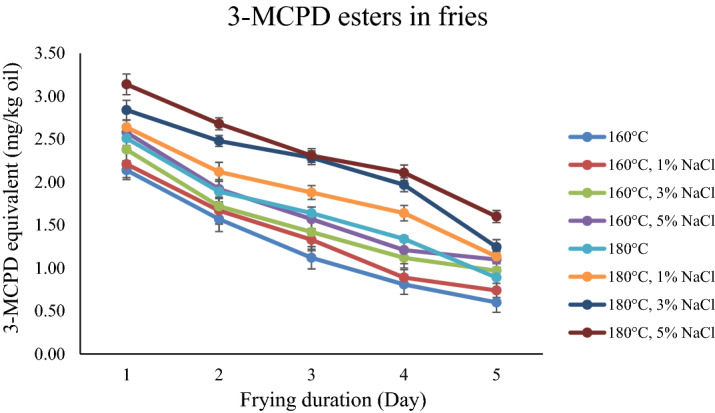
3-MCPD esters in fries in different frying systems.

The 3-monochloropropane-1,2-diol (3-MCPD) ester levels of fries prepared by frying at 160 and 180 °C with controls (distilled water) and three different salt solutions (1%, 3% and 5%) are shown in Fig. 1. The 3-MCPD esters contents ranged from 0.60 to 2.14 mg/kg oil for 160 °C and 0.89 to 2.51 mg/kg for 180 °C after each day’s frying. Generally, the 3-MCPD esters in fries under 180 °C are higher than 160 °C. The GE contents ranged from 5.23 to 6.94 mg/kg oil for 160 °C and 4.56 to 6.30 mg/kg for 180 °C after each day’s frying. For glycidyl esters (GE), the fries under 160 °C was higher as compared with fries under 180 °C. Besides that, all the oxidation parameters were increasing with the increasing of frying temperature.

### Influence of the frying duration

The influence of frying time on the level of 3-MCPD esters in fries was examined from Day 0 to Day 5. The increasing of the heating duration to Day 5 leads to the reduction of 3-MCPD esters and increase of GE. The 3-MCPD esters of fresh oil in this study was 3.5 mg/kg. The 3-MCPD esters was 0.60 mg/kg oil after 25 frying cycles at 160 °C and 0.89 mg/kg at 180 °C. In other words, the percent decreases were 82.86% for 160 °C and 74.57% for 180 °C. The GE content in the fresh oil before frying was approximately 4.0 mg/kg. The GE contents for all tested fries was 9.31 mg/kg oil after 25 frying cycles at 160 °C and 6.71 mg/kg at 180 °C. The percent increases were 73.50% for 160 °C and 57.5% for 180 °C.

In Table 1, the higher temperature frying system showed significantly higher (*p* < 0.05) FFA contents, which causes the negative effect to the taste of fries. The conjugated dienes and trienes shows the trends of increasing for both of the 160 °C and 180 °C frying systems when prolong frying duration. The lower temperature frying system showed significant (*p* < 0.05) lower p-anisidine value, representing a clear reduction in the rate of secondary oxidation due to the high frying temperature.

**Table 1:** Oxidative analyses of fries from different frying systems.

Characteristics	Day	160 °C	160 °C with 1% NaCl	160 °C with 3% NaCl	160 °C with 5% NaCl	180 °C	180 °C with 1% NaCl	180 °C with 3% NaCl	180 °C with 5% NaCl
K_232_	1	3.17 ± 0.02^e^_E_	3.25 ± 0.03^d^_D_	3.22 ± 0.02^de^_D_	3.36 ± 0.04^c^_E_	3.54 ± 0.06^bc^_E_	3.57 ± 0.15^b^_E_	3.65 ± 0.08^b^_E_	3.61 ± 0.01^a^_E_
	2	4.37 ± 0.15^d^_D_	4.34 ± 0.06^d^_C_	4.78 ± 0.07^c^_C_	4.83 ± 0.03^c^_D_	4.89 ± 0.14^c^_D_	4.95 ± 0.13^c^_D_	5.11 ± 0.01^b^_D_	5.47 ± 0.07^a^_D_
	3	5.77 ± 0.13^d^_C_	5.97 ± 0.12^ dB^	5.81 ± 0.24^ dB^	5.95 ± 0.20^d^_C_	6.45 ± 0.12^c^_C_	6.59 ± 0.04^bc^_C_	6.82 ± 0.20^b^_C_	7.12 ± 0.09^a^_C_
	4	7.06 ± 0.14^e^_B_	7.60 ± 0.11^d^_A_	7.88 ± 0.23^c^_A_	7.83 ± 0.04^bc^_B_	7.90 ± 0.13^bc^_B_	8.46 ± 0.06^b^_B_	8.52 ± 0.06^b^_B_	9.02 ± 0.14^a^_B_
	5	7.49 ± 0.04^d^_A_	7.45 ± 0.17^d^_A_	8.18 ± 0.24^c^_A_	9.36 ± 0.15^b^_A_	9.49 ± 0.16^b^_A_	9.53 ± 0.16^b^_A_	10.31 ± 0.13^a^_A_	10.57 ± 0.14^a^_A_
K_268_	1	1.60 ± 0.06^b^_C_	1.71 ± 0.11^ab^_C_	1.77 ± 0.10^a^_C_	1.81 ± 0.13^a^_C_	1.80 ± 0.12^a^_C_	1.81 ± 0.05^a^_B_	1.90 ± 0.13^a^_C_	1.92 ± 0.08^a^_D_
	2	2.00 ± 0.09^c^_BC_	2.24 ± 0.03^b^_B_	2.22 ± 0.12^b^_BC_	2.38 ± 0.11^ab^_B_	2.13 ± 0.12^bc^_BC_	2.25 ± 0.13^ab^_A_	2.30 ± 0.05^ab^_BC_	2.36 ± 0.05^a^_CD_
	3	2.12 ± 0.18^b^_B_	2.13 ± 0.09^b^_BC_	2.31 ± 0.07^ab^_B_	2.40 ± 0.04^a^_B_	2.24 ± 0.14^ab^_B_	2.27 ± 0.09^ab^_A_	2.42 ± 0.06^a^_B_	2.56 ± 0.08^a^_C_
	4	2.28 ± 0.13^b^_A_	2.38 ± 0.10^ab^_A_	2.47 ± 0.10^ab^_A_	2.45 ± 0.04^ab^_B_	2.38 ± 0.13^ab^_B_	2.47 ± 0.17^ab^_A_	2.58 ± 0.12^a^_A_	2.64 ± 0.08^a^_B_
	5	2.32 ± 0.16^b^_A_	2.41 ± 0.18^ab^_A_	2.45 ± 0.14^ab^_B_	2.61 ± 0.09^a^_A_	2.45 ± 0.12^ab^_A_	2.48 ± 0.20^ab^_A_	2.53 ± 0.09^ab^_A_	2.78 ± 0.08^a^_A_
p-anisidine value (pAV)	1	28.54 ± 1.72^de^_E_	31.82 ± 1.01^c^_E_	32.89 ± 1.06^bc^_E_	35.23 ± 1.24^a^_E_	34.48 ± 0.82^b^_E_	21.05 ± 1.84^f^_E_	26.36 ± 1.01^e^_D_	28.29 ± 0.79^d^_D_
	2	50.42 ± 1.31^d^_D_	52.23 ± 1.46^c^_D_	52.98 ± 1.22^b^_D_	54.32 ± 1.62^b^_D_	58.72 ± 1.11^ab^_D_	60.13 ± 1.24^ab^_D_	59.15 ± 1.33^ab^_C_	64.49 ± 1.17^a^_C_
	3	65.78 ± 1.47^d^_C_	67.81 ± 0.98^c^_C_	68.41 ± 1.15^b^_C_	69.24 ± 1.52^b^_C_	73.32 ± 1.32^ab^_C_	74.96 ± 0.88^ab^_C_	79.87 ± 0.93^a^_B_	81.10 ± 1.62^a^_B_
	4	71.92 ± 1.46^c^_B_	72.02 ± 0.70^c^_B_	71.60 ± 1.50^c^_B_	72.90 ± 1.34^c^_B_	77.89 ± 0.86^bc^_B_	79.17 ± 1.22^b^_B_	81.27 ± 1.52^a^_B_	83.51 ± 1.51^a^_B_
	5	77.18 ± 1.78^c^_A_	80.95 ± 1.39^bc^_A_	81.49 ± 1.83^bc^_A_	82.98 ± 1.61^b^_A_	81.69 ± 2.14^bc^_A_	82.97 ± 2.18^b^_A_	85.33 ± 1.36^a^_A_	88.53 ± 2.17^a^_A_
Free fatty acid (FFA) (%)	1	0.12 ± 0.01^f^_E_	0.16 ± 0.00^e^_E_	0.19 ± 0.00^d^_E_	0.21 ± 0.00^b^_E_	0.16 ± 0.00^e^_E_	0.19 ± 0.00^d^_E_	0.20 ± 0.00^c^_E_	0.22 ± 0.00^a^_E_
	2	0.16 ± 0.00^g^_D_	0.19 ± 0.00^f^_D_	0.23 ± 0.00^d^_D_	0.27 ± 0.00^b^_D_	0.19 ± 0.00^f^_D_	0.21 ± 0.00^e^_D_	0.24 ± 0.00^c^_D_	0.28 ± 0.00^a^_D_
	3	0.19 ± 0.00^g^_C_	0.22 ± 0.00^f^_C_	0.27 ± 0.00^d^_C_	0.28 ± 0.00^c^_C_	0.22 ± .000^f^_C_	0.26 ± 0.00^e^_C_	0.32 ± 0.00^b^_C_	0.38 ± 0.00^a^_C_
	4	0.23 ± 0.00^f^_B_	0.31 ± 0.01^c^_B_	0.28 ± 0.00^ dB^	0.31 ± 0.00^c^_B_	0.25 ± 0.01^e^_B_	0.28 ± 0.00^ dB^	0.34 ± 0.00^b^_B_	0.41 ± 0.00^a^_B_
	5	0.25 ± 0.00^g^_A_	0.39 ± 0.01^c^_A_	0.34 ± 0.01^d^_A_	0.35 ± 0.01^d^_A_	0.28 ± 0.00^f^_A_	0.31 ± 0.00^e^_A_	0.56 ± 0.01^a^_A_	0.52 ± 0.00^b^_A_

Each value in the table represents the mean ± standard deviation of six analyses from two replications. Means within each row with different superscripts are significantly (*p* < 0.05) different. Means within each column with different subscripts are significantly (*p* < 0.05) different.

### Influence of the salt (NaCl)

**Figure 2:**
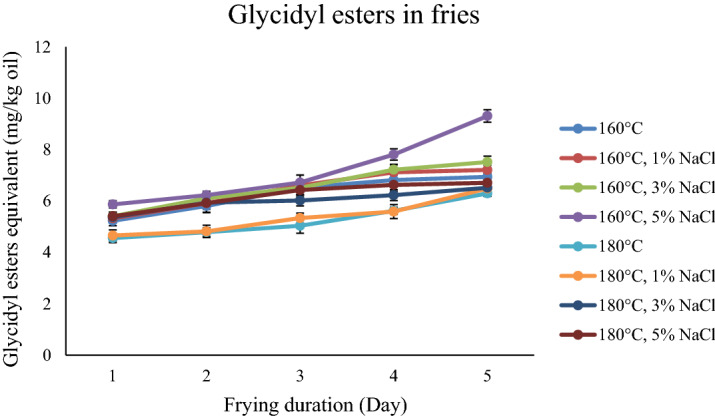
Glycidyl esters in fries in different frying systems.

Figure 1 shows the content of 3-MCPD with the influence of salt. When the concentration of salt was increasing, the content of 3-MCPD esters were significantly (*p* < 0.05) increased. This finding is in agreement with another study that reported that the generation of 3-MCPD esters increased with the increasing sodium chloride concentration^12^. The influences of salt on the GE in different frying systems are shown in Fig. 2. There was some increase in the GE with the increased concentration of salt. However, there was no significant difference (*p* > 0.05) between different concentrations of salt. The increases of p-anisidine, CD and CT were observed when the amount of salt increased.

## Discussion

The formation of 3-monochloropropane-1,2-diol (3-MCPD) esters and glycidyl esters (GE) are a multivariate problem, which is affected by fat and salt content, processing temperature and time. This study is focused on the content of 3-MCPD esters and GE in fries which undergone different frying parameters. The frying oil was absorbed in the fried potatoes during frying. The content of lipids in the fried potatoes was approximately 40%. Fresh potatoes are mainly made up of water, with an average content of 75%. During the frying, the high temperatures result in a partial evaporation of the moisture in the fries, and travels through the surrounding oil. The oil replaces the water lost in fries. A higher frying temperature under atmospheric conditions causes more oil to accumulate in the fries. This is because the higher frying temperature leads to the rapid formation of a solid crust, which is favorable for oil absorption^13^. Therefore, it is necessary to monitor the amounts of 3-MCPD esters and GE in fries during repeated frying.

In this study, the temperature of 160 °C and 180 °C were selected because they are commonly used deep frying temperatures. A higher temperature results in a higher 3-MCPD esters level in the fries, in agreement with other studies^14^, but the degree of increase was not the same for the different frying parameters, which was attributed to the different contents of salt and frying temperatures. For 3-MCPD esters, the rate of degradation is lower than the formation rate under high temperatures. It is believed that the level of GE is affect by the competitive reactions between generation and decomposition^15^. In contrast with 3-MCPD, for GE, the rate of degradation is higher than the formation rate under high temperatures.

Five days frying durations did not contribute to significant differences in the acylglycerol composition. The longer the heating time, the more 3-MCPD esters were degraded. The heat of frying reduces, oxidizes, and degrades the generated 3-MCPD esters during the process. The GE was stable under a long frying duration at a constant heating temperature, and therefore the content of GE gradually increased.

The mechanisms of 3-MCPD esters and GE are little understood, but it is commonly agreed that acylglycerols are one of the precursors. A few studies^9,16,17^ have suggested that the formation pathway of 3-MCPD esters is involved TAG. In our study the frying systems with 180 °C, TAG content was lower than the frying systems with 160 °C. This shows that more TAG was used up more for the 3-MCPD formation under higher temperature, in this study which is 180 °C. The MAG and DAG were involved in the formation of GE. Therefore, the MAG and DAG of the lower temperature frying systems were significantly lower as shown in Table 2.

**Table 2:** Acylglycerol composition of fries in different frying systems.

Acylglycerol composition (%)	Day	160 °C	160 °C with 1% NaCl	160 °C with 3% NaCl	160 °C with 5% NaCl	180 °C	180 °C with 1% NaCl	180 °C with 3% NaCl	180 °C with 5% NaCl
Free fatty acid/monoacylglycerol (FFA/MAG)	1	0.11 ± 0.07^b^_A_	0.13 ± 0.04^b^_A_	0.13 ± 0.03^b^_A_	0.14 ± 0.03^b^_B_	0.21 ± 0.04^ab^_C_	0.22 ± 0.06^ab^_B_	0.23 ± 0.02^a^_B_	0.25 ± 0.06^a^_B_
	2	0.13 ± 0.07^b^_A_	0.15 ± 0.06^ab^_A_	0.15 ± 0.03^b^_A_	0.19 ± 0.03^ab^_AB_	0.25 ± 0.05^ab^_B_	0.28 ± 0.05^a^_AB_	0.28 ± 0.07^a^_AB_	0.32 ± 0.07^a^_AB_
	3	0.14 ± 0.08^b^_A_	0.15 ± 0.05^b^_A_	0.17 ± 0.09^ab^_A_	0.20 ± 0.05^b^_AB_	0.29 ± 0.05^a^_B_	0.31 ± 0.05^a^_AB_	0.32 ± 0.05^a^_A_	0.33 ± 0.08^a^_AB_
	4	0.16 ± 0.05^b^_A_	0.17 ± 0.06^b^_A_	0.17 ± 0.05^b^_A_	0.26 ± 0.07^ab^_A_	0.31 ± 0.04^a^_AB_	0.34 ± 0.07^a^_AB_	0.33 ± 0.07^a^_A_	0.34 ± 0.08^a^_AB_
	5	0.16 ± 0.01^c^_A_	0.18 ± 0.1^c^_A_	0.22 ± 0.12^b^_A_	0.35 ± 0.07^a^_A_	0.37 ± 0.01^a^_A_	0.37 ± 0.06^a^_A_	0.39 ± 0.03^a^_A_	0.39 ± 0.04^a^_A_
Diacylglycerol (DAG)	1	2.32 ± 0.34^b^_B_	2.54 ± 0.76^ab^_A_	2.65 ± 0.51^ab^_B_	2.98 ± 0.36^ab^_B_	3.10 ± 0.46^ab^_B_	3.32 ± 0.72^ab^_A_	3.58 ± 0.29^a^_B_	3.46 ± 0.40^a^_C_
	2	2.63 ± 0.46^b^_AB_	2.70 ± 0.44^b^_A_	2.68 ± 0.63^ab^_B_	3.27 ± 0.49^ab^_B_	3.83 ± 0.57^a^_AB_	4.02 ± 0.75^a^_A_	3.99 ± 0.31^a^_AB_	4.06 ± 0.35^a^_BC_
	3	3.04 ± 0.61^b^_AB_	2.91 ± 0.65^b^_A_	2.92 ± 0.31^b^_B_	3.45 ± 0.61^ab^_B_	3.85 ± 0.39^ab^_AB_	4.01 ± 0.52^ab^_A_	4.36 ± 0.94^a^_AB_	4.55 ± 0.61^a^_AB_
	4	3.33 ± 0.37^b^_A_	3.41 ± 0.84^b^_A_	3.64 ± 0.21^b^_A_	3.87 ± 0.53^ab^_AB_	3.91 ± 0.49^ab^_AB_	4.15 ± 0.12^ab^_A_	4.39 ± 0.43^ab^_A_	4.68 ± 0.37^a^_B_
	5	3.58 ± 0.54^b^_A_	3.77 ± 0.54^ab^_A_	3.85 ± 0.54^ab^_AB_	4.33 ± 0.24^ab^_A_	4.01 ± 0.43^ab^_A_	4.37 ± 0.60^ab^_A_	5.05 ± 0.77^a^_A_	5.47 ± 0.33^a^_A_
Triacylglycerol (TAG)	1	97.56 ± 0.26^a^_A_	97.33 ± 0.71^ab^_AB_	97.21 ± 0.62^ab^_A_	96.87 ± 0.34^b^_A_	96.69 ± 0.38^b^_A_	96.46 ± 0.66^b^_A_	96.19 ± 0.47^b^_A_	96.29 ± 0.34^b^_A_
	2	97.24 ± 0.39^a^_AB_	97.15 ± 0.38^a^_A_	97.16 ± 0.61^a^_A_	96.54 ± 0.46^ab^_A_	95.91 ± 0.48^b^_AB_	95.69 ± 0.77^b^_A_	95.73 ± 0.33^b^_A_	95.62 ± 0.48^b^_A_
	3	96.83 ± 0.52^a^_AB_	96.94 ± 0.61^a^_AB_	96.90 ± 0.22^a^_A_	96.35 ± 0.90^ab^_AB_	95.86 ± 0.34^b^_B_	95.68 ± 0.47^b^_A_	95.31 ± 0.89^b^_B_	95.12 ± 0.50^b^_AB_
	4	96.50 ± 0.42^a^_B_	96.43 ± 0.36^a^_AB_	96.18 ± 0.60^a^_A_	95.87 ± 0.83^ab^_AB_	95.78 ± 0.45^ab^_B_	95.51 ± 0.43^ab^_A_	95.27 ± 0.31^b^_AB_	94.98 ± 0.53^b^_AB_
	5	96.26 ± 0.67^a^_B_	96.05 ± 0.57^a^_B_	95.93 ± 0.90^a^_A_	95.32 ± 0.67^ab^_B_	95.62 ± 0.43^a^_B_	95.26 ± 0.54^a^_A_	94.56 ± 0.75^ab^_B_	94.14 ± 0.57^b^_B_

Each value in the table represents the mean ± standard deviation of six analyses from two replications. Means within each row with different superscripts are significantly (*p* < 0.05) different. Means within each column with different subscripts are significantly (*p* < 0.05) different.

Besides that, the oxidative changes of the fries were monitored. Acid value is not recommended for the measurement of oxidation level of frying oil. This is because peroxides are destroyed under high temperature, and the new peroxides formed when the temperature going down. However, acid value can be a good indicator for off-flavor^18^. Conjugated dienes (CD) and trienes (CT) are better measurements of oxidation because they remain stable compared with the peroxide value. P-anisidine and conjugated dienes showed good relation with total polar content (TPC) as reported earlier^19^. The analysis method of p-anisidine and CD are simpler and more rapid as compared to TPC. Therefore, p-anisidine and conjugated dienes has been used in this study to monitor the oxidation condition of the frying oil. The result in this study is in accordance with the finding by Houhoula, et al.^20^ that p-anisidine and conjugated dienes increased with the increasing of frying duration^19^. Conjugated trienes shows increasing trend as well, but in lower value. This shows that some of the primary oxidation products has change to the secondary oxidation products.

Chloride ion is the key factor in the formation pathway of 3-MCPD esters, no matter naturally presents or added intentionally^21^. Sodium chloride is commonly used as a seasoning in making potato chips to regulate the taste. Hence, salt was selected as one of the factors in this study. Chlorine donors are formed and decomposed during the heat treatment. The reactions of chlorine ions with the palm olein frying oil are highly complex and likely to happen both via direct routes and indirectly via intermediates^22^. This means that the presence of chloride ion in frying system had no significant effect on the concentration of GE. This is because sodium can act as pro-oxidant. Besides that, the trace amount of iron and copper in edible salt is active pro-oxidant too.

## Methods

### Materials and chemicals

The frying oil, Refined Bleached Deodorized palm olein was purchased from a Malaysia oil production company (Selangor, Malaysia). Potatoes (Russet var.) that used to prepare potato chips were obtained from a local market. The 3-MPCD and GE standards and internal standards were bought from Toronto Research Chemicals, Inc. (Toronto, Canada). The solvents were from Merck (Darmstadt, Germany).

Potato chips of 2 mm thickness were prepared from fresh potatoes using a hand slicer. The potato chips were soaked in distilled water or a salt solution (1, 3 and 5%). The salt used was sodium chloride (NaCl). Before frying, the potato chips were blotted on tissue paper and weighed into each batches of 100 g.

### Experiment methods

#### Preparation of fries

There was a total of eight frying parameters, with different combination of temperature (160 °C and 180 °C) and salt concentration (0%, 1%, 3% and 5%). The frying with each set of parameters was duplicated using an electric deep fryer (Cornell, Malaysia).

The frying was started with 3 kg of RBD palm olein. The temperature was raised and hold at the target temperature for 10 min. Fifty milliliters of oil sample was kept before heating. 100 g of fresh potato chips were fried for 2.5 min and followed with 17.5 min intervals for 5 cycles every day. It was conducted for 5 days to comprise a total of 25 frying cycles. There was no oil replenishment for the 5 days frying. The fryer was uncovered when the frying was carried out. After the end of 5th cycle of the day, 50 mL of oil was transferred to an amber glass bottle and the fryer was turned off. Then, the oil was kept in the electric fryer, and continue at the next day. Lastly, all the collected oil samples were kept in chiller at − 20 °C until analysed.

#### Oil extraction

The oil from fries was extracted by an automated fat extractor, Soxtec 8000 (Foss Analytical AB, Sweden, Denmark). Prior to extraction, 10 g of fries was cut and dry in an oven for 2 h at 80 ºC. After that, dried fries was put in the thimble and cover with cotton wool. Each extraction cup was filled with 80 mL of *n*-hexane. The extraction program was started with 20 min boiling, subsequently followed by 40 min of rinsing, and lastly 10 min of recovery to remove the solvent.

#### Conjugated dienes (K_232_) and trienes (K_268_)

AOCS Method Ch 5-91 was used to determine the conjugated dienes and trines. The oil samples were prepared with isooctane and the absorbance at 232 nm for dienes and 268 nm for trienes were measured.

#### P-anisidine value

AOCS Official Method Cd 18-90 was used to determine the p-Anisidine value.

#### Free fatty acid content

AOCS Method Ca 5a-40 was used to determine the free fatty acids (FFA). The data were presented in palmitic acid percentage.

#### Acylglycerol composition

The acylglycerol compositions in fries were analyzed using an Alliance model Waters 22695 Separation Module RP-HPLC system equipped with an evaporative light scattering detector (model 242) as described by Wong et al.^23^. A Merck silica capillary column (Darmstadt, Germany; Purospher STAR RP-18, 250 mm × 4.6 mm × 5 μm) was used for separation. Before the analysis, oil samples in 10 mg/mL were prepared with acetone. After that, the sample solutions were pass through a PTFE 0.45 μm filter. To determine acylglycerol compositions, a dual-solvent gradient was used, where solvent A was acetone and solvent B was acetonitrile. The column was developed at a 1 mL/min gradient flow with 15 min of 1:1 A/B to 5.5:4.5, 15 min to 6:4 A/B, and 15 min to 6.5:3.5 A/B. The column temperature was 35 °C. The nebulizer temperature was programmed at 36 °C with nitrogen gas at 35 psi. The drift tube temperature was 45 °C. FFA/MAG were monitored at 3 to 5 min, DAG at 6–12 min and lastly TAG at 20–38 min.

#### Determination of 3-MCPD esters and glycidyl esters

The modified AOCS method Cd 29a-13 was used. The 3-MCPD and glycidol contents of the oil samples were analyzed using a gas chromatography—mass spectrometry (5975C Agilent Technologies, USA). The separation capillary column used was HP-5MS capillary column (Agilent Technologies, USA) with 30 m length, 0.25 mm internal diameter and the film thickness of 0.25 µm. The injector was set at splitless mode at 250 °C. Besides, the column temperature was started from 80 °C for 1 min and increased to 170 °C at 10 °C/min, to 200 °C at 3 °C/min and lastly to 300 °C at 15 °C/min and holding for 15 min. Helium (0.8 mL/min) was used as the carrier gas. A 1 µl sample was injected. In the quantitative analysis of the derivatized 3-MCPD, the quantifier ions were monitored at m/z 147 for 3-MCPD and m/z 150 for 3-MCPD-d_5_. The qualifiers for 3-MCPD were ions at m/z 196 and 198, and for 3-MCPD-d_5_ were m/z 201. In the quantitative analysis of the 3-MBPD compound, the quantifier ions were m/z 147 for 3-MBPD and m/z 150 for 3-MBPD-d_5_. For the qualifiers, 3-MBPD was monitored at m/z 240 and 3-MBPD-d_5_ at m/z 245.

### Statistical analysis

Two parallel examinations were made of each sample, and the measurements were duplicated twice. All the results were presented as the mean ± SD. One way analysis of variance (ANOVA) was used to compared between the means. A probability value of 95% (*p* < 0.05) was used to demonstrate the significance.

## Conclusions

Our study found that the effects of deep-fat frying on potato chips were in order of sodium chloride < frying duration < frying temperature. A high frying temperature resulted in high 3-MCPD esters but lower GE. A long frying duration caused a reduction of 3-MCPD esters and an increase in GE. The soaking of potato chips in salt solution significantly increases 3-MCPD esters, but no significant difference was observed in the GE content. The qualities of fries were fall within the safety range.

## References

[1] Moumtaz S (2019). Toxic aldehyde generation in and food uptake from culinary oils during frying practices: peroxidative resistance of a monounsaturate-rich algae oil. Sci. Rep..

[2] Zhang Q, Saleh AS, Chen J, Shen Q (2012). Chemical alterations taken place during deep-fat frying based on certain reaction products: a review. Chem. Phys. Lipids.

[3] Díaz-Sánchez F, Santos E, Sosa M, Ramírez-Corona N (2019). Stability of Palm olein with or withoUt antioxidants dUring indUstrial ContinUoUs deeP-fat frying of wheat snaCks. J. Oil Palm Res..

[4] Aniolowska M, Kita A (2016). Monitoring of glycidyl fatty acid esters in refined vegetable oils from retail outlets by LC-MS. J. Sci. Food Agric..

[5] Yao Y (2019). Molecular reaction mechanism for the formation of 3-chloropropanediol esters in oils and fats. J. Agric. Food Chem..

[6] Choe E, Choe E, Min DB (2007). Chemistry of deep-fat frying oils. J. Food Sci..

[7] Haines TD (2011). Direct determination of MCPD fatty acid esters and glycidyl fatty acid esters in vegetable oils by LC-TOFMS. J. Am. Oil Chem. Soc..

[8] Kamikata K (2019). Occurrence of 3-MCPD, 2-MCPD and glycidyl esters in extra virgin olive oils, olive oils and oil blends and correlation with identity and quality parameters. Food Control.

[9] Weißhaar R, Perz R (2010). Fatty acid esters of glycidol in refined fats and oils. Eur. J. Lipid Sci. Technol..

[10] Ilko V, Zelinková Z, Doležal M, Velíšek J (2011). 3-Chloropropane-1,2-diol fatty acid esters in potato products. Czech J. Food Sci..

[11] Tian X, Fisk ID (2012). Salt release from potato crisps. Food Funct..

[12] Hamlet CG (2011). Formation and occurrence of esters of 3-chloropropane-1,2-diol (3-CPD) in foods: what we know and what we assume. Eur. J. Lipid Sci. Technol..

[13] Garayo J, Moreira R (2002). Vacuum frying of potato chips. J. Food Eng..

[14] Rahn AKK, Yaylayan VA (2011). What do we know about the molecular mechanism of 3-MCPD ester formation?. Eur. J. Lipid Sci. Technol..

[15] Shimizu M, Weitkamp P, Vosmann K, Matthäus B (2013). Influence of chloride and glycidyl-ester on the generation of 3-MCPD- and glycidyl-esters. Eur. J. Lipid Sci. Technol..

[16] Destaillats F, Craft BD, Dubois M, Nagy K (2012). Glycidyl esters in refined palm (*Elaeis guineensis*) oil and related fractions. Part I: formation mechanism. Food Chem..

[17] Craft BD, Nagy K, Sandoz L, Destaillats F (2012). Factors impacting the formation of monochloropropanediol (MCPD) fatty acid diesters during palm (*Elaeis guineensis*) oil production. Food Addit. Contam. Part A Chem. Anal. Control Expo. Risk Assess..

[18] Farhoosh R, Khodaparast MHH, Sharif A, Rafiee SA (2012). Olive oil oxidation: rejection points in terms of polar, conjugated diene, and carbonyl values. Food Chem..

[19] Farhoosh R, Moosavi SMR (2009). Evaluating the performance of peroxide and conjugated diene values in monitoring quality of used frying oils. mdrsjrns.

[20] Houhoula DP, Oreopoulou V, Tzia C (2002). A kinetic study of oil deterioration during frying and a comparison with heating. J. Am. Oil. Chem. Soc..

[21] Freudenstein A, Weking J, Matthäus B (2013). Influence of precursors on the formation of 3-MCPD and glycidyl esters in a model oil under simulated deodorization conditions. Eur. J. Lipid Sci. Technol..

[22] Nagy K, Sandoz L, Craft BD, Destaillats F (2011). Mass-defect filtering of isotope signatures to reveal the source of chlorinated palm oil contaminants. Food Addit. Contam. Part A Chem. Anal. Control Expo. Risk Assess..

[23] Wong YH (2017). Factors impacting the formation of 3-MCPD esters and glycidyl esters during deep fat frying of chicken breast meat. J. Am. Oil. Chem. Soc..

